# Disrupted dynamic network reconfiguration of the brain functional networks of individuals with autism spectrum disorder

**DOI:** 10.1093/braincomms/fcac177

**Published:** 2022-08-01

**Authors:** Min Wang, Lingxiao Wang, Bo Yang, Lixia Yuan, Xiuqin Wang, Marc N Potenza, Guang Heng Dong

**Affiliations:** Center for Cognition and Brain Disorders, School of Clinical Medicine and the Affiliated Hospital of Hangzhou Normal University, Hangzhou, Zhejiang Province 311121, PR China; Center for Cognition and Brain Disorders, School of Clinical Medicine and the Affiliated Hospital of Hangzhou Normal University, Hangzhou, Zhejiang Province 311121, PR China; Center for Cognition and Brain Disorders, School of Clinical Medicine and the Affiliated Hospital of Hangzhou Normal University, Hangzhou, Zhejiang Province 311121, PR China; Center for Cognition and Brain Disorders, School of Clinical Medicine and the Affiliated Hospital of Hangzhou Normal University, Hangzhou, Zhejiang Province 311121, PR China; Zhejiang Key Laboratory for Research in Assessment of Cognitive Impairments, Hangzhou, Zhejiang Province 310000, PR China; Center for Cognition and Brain Disorders, School of Clinical Medicine and the Affiliated Hospital of Hangzhou Normal University, Hangzhou, Zhejiang Province 311121, PR China; Zhejiang Key Laboratory for Research in Assessment of Cognitive Impairments, Hangzhou, Zhejiang Province 310000, PR China; Department of Psychiatry and Child Study Center, Yale University School of Medicine, New Haven, CT 201942, USA; Connecticut Mental Health Center, New Haven, CT 201942, USA; Connecticut Council on Problem Gambling, Wethersfield, CT 201942, USA; Department of Neuroscience and Wu Tsai Institute, Yale University, New Haven, CT 201942, USA; Center for Cognition and Brain Disorders, School of Clinical Medicine and the Affiliated Hospital of Hangzhou Normal University, Hangzhou, Zhejiang Province 311121, PR China; Zhejiang Key Laboratory for Research in Assessment of Cognitive Impairments, Hangzhou, Zhejiang Province 310000, PR China

**Keywords:** ASD, community structure, dynamic network, default mode network, basal ganglia network

## Abstract

Human and animal studies on brain functions in subjects with autism spectrum disorder have confirmed the aberrant organization of functional networks. However, little is known about the neural features underlying these impairments. Using community structure analyses (recruitment and integration), the current study explored the functional network features of individuals with autism spectrum disorder from one database (101 individuals with autism spectrum disorder and 120 healthy controls) and tested the replicability in an independent database (50 individuals with autism spectrum disorder and 74 healthy controls). Additionally, the study divided subjects into different age groups and tested the features in different subgroups. As for recruitment, subjects with autism spectrum disorder had lower coefficients in the default mode network and basal ganglia network than healthy controls. The integration results showed that subjects with autism spectrum disorder had a lower coefficient than healthy controls in the default mode network–medial frontal network and basal ganglia network–limbic networks. The results for the default mode network were mostly replicated in the independent database, but the results for the basal ganglia network were not. The results for different age groups were also analysed, and the replicability was tested in different databases. The lower recruitment in subjects with autism spectrum disorder suggests that they are less efficient at engaging these networks when performing relevant tasks. The lower integration results suggest impaired flexibility in cognitive functions in individuals with autism spectrum disorder. All these findings might explain why subjects with autism spectrum disorder show impaired brain networks and have important therapeutic implications for developing potentially effective interventions.

## Teaser

Using community structure analyses, the autism spectrum disorder subjects showed lower coefficient in default mode network (DMN) and basal ganglia network. The results in DMN were also observed in different age groups and replicated in different databases.

## Graphical Abstract

**Graphical Abstract:**
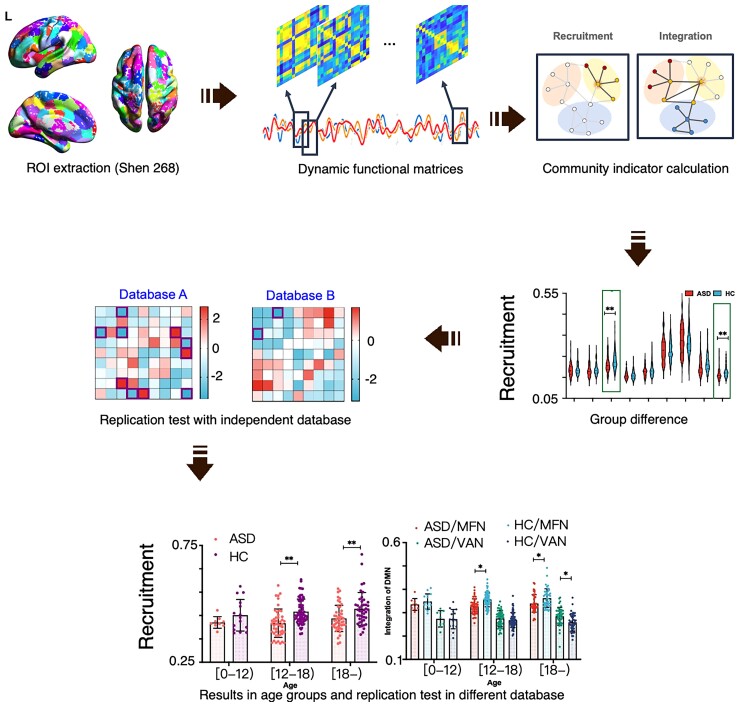

## Introduction

Autism spectrum disorder (ASD) symptoms are usually defined as social communication difficulties, restricted interests and repetitive behaviours.^1–3^ Children with ASD show a reduced preference for social stimuli,^3–5^ diminished interest in collaborative social activities,^6,7^ deficits in reciprocal social interaction^2,8^ and receive less pleasure from social situations^9,10^ than their typically developing peers. Multiple differences in functional and morphological brain phenotypes have been reported in subjects with ASD,^1,11–13^ including altered brain development in functional responses, regional cortical thickness, surface area and structure volume.^13,14^ Additionally, specific brain regions were found to be associated with functional changes during cognitive processes, such as when facing social cues, cravings or executive functions.^15,16^ Most of these changes are associated with individual features, such as symptom severity, age and sex.

Recent advances in the brain sciences have suggested that cognitive function is accomplished by interactions of the brain network rather than by a specific region.^17,18^ For ASD, studies found alterations in the default mode network (DMN), atypical social brain networks and basal ganglia network (BGN) compared with healthy controls (HCs),^19–22^ and these features were associated with their behavioural performances. For example, subjects with ASD show enhanced DMN connectivity compared with HCs, which may be related to their lack of verbal and nonverbal communication.^23^ A lower strength of posterior cingulate cortex–medial prefrontal cortex connectivity in subjects with ASD is associated with poorer social functioning.^24^ The strength of intramodule connectivity was significantly lower in the DMN and revealed a strong correlation with language.^25^ Both structural and functional circuit aberrations in the mesolimbic reward pathway are related to parent-reported measures of impaired social interactions in affected children.^19^

Although studies have reported great progress in understanding the functional networks in individuals with ASD, limitations also existed in these studies. First, although studies have depicted brain network features in individuals with ASD, the neural structures underlying these features remain unclear. Exploring this issue is important to understand the neural underpinnings of ASD and is valuable for the development of potential intervention strategies. Second, most of these studies hypothesized that the interactive connection between brain regions/networks throughout the whole process was temporally stationary.^26–28^ However, the human brain is a complex system; the functional connectivity of the human brain changes during different cognitive processes, such as learning, growth and even rest.^29,30^ The brain dynamically integrates and coordinates the interactions of different brain areas to complete complex cognitive functions.

Community structure is a functionally relevant graph metric used to study the organization of functional systems in brain networks.^31^ Recently, several prospective ASD studies illustrated the community variability at node- and global-level. For instance, Harlalka *et al*.^32^ reported significantly higher dynamic variability in functional connection in ASD, as well as a positive correlation of symptom severity with flexibility of sensorimotor and visual regions. Xie *et al*.^33^ indicated that ASD showed a higher whole-brain mean and lower standard deviation in global module dynamics, associated with previously identified autism-related genes. The above findings well illustrate the global or local properties of ASD's community dynamics. However, for the network level, these studies generalize network variability from node differences in a descriptive manner, which may involve excessive inferences of networks. In addition, the dynamic community metrics, e.g. connectivity variability and flexibility primarily detect the frequency of changes in the community affiliation of nodes across time windows, providing inferences on the discrete properties within a single network. However, how the process of segregation and integration of brain networks was represented in ASD remains unclear.

In general, the interactive connections within community nodes (or brain regions) are strong and dense, whereas interactive connections between communities are sparse.^34^ Thus, two indices are available to measure the community features: recruitment, which refers to the probability that a brain region is in the same community as other nodes from its own network; and integration, which refers to the probability that a brain region is in the same community as nodes from other networks.^35^ The two can delineate well the functional segregation and integration of whole-brain networks. Researchers have identified community structure in both structural and functional networks in the healthy human brain.^36^ In functional imaging studies, especially fMRI, researchers use blood oxygen level-dependent signals to analyse fluctuations in brain activities, which are sufficient to study the dynamic properties of brain networks.^37^ This method is increasingly used in psychiatric studies, for example, in patients with schizophrenia,^38^ unipolar depression,^39^ major depressive disorder,^40^ and temporal lobe epilepsy.^35^ These studies provide insights into the neural features of these psychiatric disorders.

The primary aim of the current study was to address the limitations of previous studies on ASD and provide a better understanding of the neural features underlying altered brain functional networks in individuals with ASD. We examined the distribution of community assignment across the entire scanning time and compared the coupling changes in the community structure of the brain functional networks in subjects to achieve this goal. We hypothesized that patients with ASD would have a different community structure than the HC group. Second, as studies have revealed that functional brain network features change with growth, we decided to explore whether we would observe similar dynamic community features in different age groups in the current study.

## Methods and procedures

### Participants

The dataset in this study originated from the initial Autism Brain Imaging Data Exchange (ABIDE I and ABIDE II). ABIDE I involved 24 international sites with 1112 subjects, including 539 patients with ASD and 573 HCs (ages 7–64 years, median of 14.7 years across groups). ABIDE II was used as independent data to test the replicability of the results from ABIDE I. Functional and structural brain imaging datasets and phenotypic datasets were downloaded from the ABIDE website (http://fcon_1000.projects.nitrc.org/indi/abide/).

We used strict inclusion and exclusion criteria when selecting subjects to satisfy the requirements of the community structure analysis. The detailed inclusion and exclusion criteria are shown in the Supplementary files. Finally, 221 subjects (101 individuals with ASD and 120 HCs) from ABIDE I were selected in the current study. Twenty-three ASD participants and 1 HCs reported a history of medication or were receiving treatment. In addition, 124 subjects (50 individuals with ASD and 74 HCs) from ABIDE II were selected for the current study. Four ASD participants reported a history of medication. Data from ABIDE I were analysed in the current analyses, and the data from ABIDE II were used for replication testing. All demographic information for participants in ABIDE I and ABIDE II is shown in Table 1.

**Table 1:** Demographic information of the subjects included in current study

Subject group	Age	Sex	FIQ	VIQ	PIQ
ABIDE-I					
Controls (*n* = 120)	17.7 ± 5.7	101/19	110.6 ± 10.9		
ASD(*n* = 101)	18.1 ± 6.1	90/11	105.1 ± 16.6		
ABIDE-II					
Controls(*n* = 74)	20.8 ± 6.7	65/9	117.5 ± 12.3	115.9 ± 13.1	115.1 ± 14.3
ASD(*n* = 50)	19.0 ± 8.9	43/7	111.8 ± 13.9	110.9 ± 16.3	108.3 ± 14.8

FIQ, full scale intelligence quotient; VIQ, verbal IQ; PIQ, performance IQ.

### MRI data preprocessing

**Figure 1:**
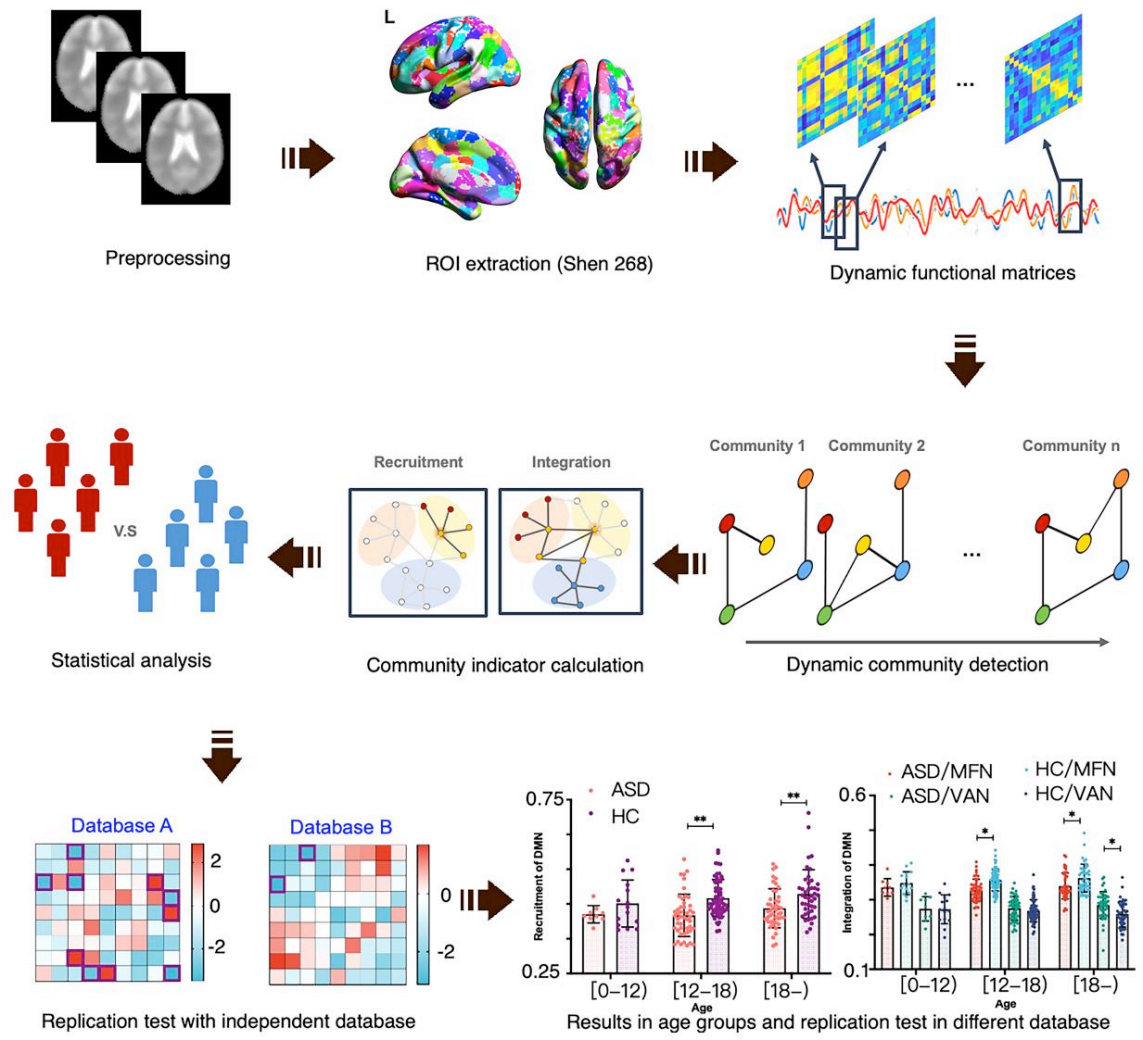
**The whole data analysis procedure used in the current study**. This figure shows the whole process from preprocessing to statistical analyses and replication testing.

All the preprocessing and analysis steps are depicted in Fig. 1. Data preprocessing were performed with DPABI V5.1 (http://rfmri.org/dpabi) and SPM12 (http://www.fil.ion.ucl.ac.uk/spm/software/spm12/). Preprocessing steps included: (i) discarding the first 10 time points; (ii) slice timing correction; (iii) head motion correction and scrubbing; (iv) spatial normalization with the forward transformation field from the unified segmentation of anatomic images, and subsequent resampling into 3 mm × 3 mm × 3 mm; (v) spatial smoothing with a 3D isotropic Gaussian kernel with a full width at half maximum of 6 mm; (vi) removing the linear trend; (vii) global signal and nuisance covariates regression, including head motion covariates with Friston 24-parameter model and white matter and cerebrospinal fluid signal; and (viii) filtering the data with a passband filter of 0.01–0.08 Hz.

### Network construction

Dynamic network calculation was performed with Gretna toolbox (https://www.nitrc.org/projects/gretna/). When constructing the brain networks, we used Shen’s brain atlas, which consists of 268 regions of interest (ROIs) of 3 mm dimensions and provides whole-brain coverage of the cerebral cortex and cerebellum.^41^ Cerebral networks were computed for each participant, and BOLD signals were extracted from each ROI during the whole scan.

Since the brain and the cerebellum were relatively physically independent, we excluded the ROIs within the cerebellum and assigned the remaining 219 ROIs to nine functional modules corresponding to the DMN, medial frontal network (MFN), frontoparietal network (FPN), limbic network, motor network, visual Networks I and II, visual association network, and BGN. We used this modular structure to compute the graph theory measures described below.

The time-series data were then split into a consecutive series of time windows with a length of 20 TR and overlapping with contiguous windows by 50%. The dynamic functional connectivity between each pair of ROIs was estimated, generating a 219 × 219 adjacency matrix for each time window. All negative connections were set to 0, as their representation in the network remains unclear. Finally, the adjacency matrices in all time windows were linked to form a multilayer network.

### Multilayer community detection

Multilayer community detection was performed with GenLouvain toolbox (https://github.com/GenLouvain/GenLouvain). A community describes a group of nodes that are more strongly connected to each other than to nodes outside of their community,^42^ whereas a multilayer community further characterizes their reconfiguration over time. In the current study, we used a generalized Louvain community detection algorithm^31^ involving the following multilayer modularity quality function:

(1) $$Q\;=\frac{1}{2\mu }\sum_{ijlr}\;[(A_{ijl\;}-\;\gamma_{l}V_{ijl})\delta_{lr}+\;\delta_{ij}\omega_{\;jlr}]\delta (g_{il},\;g_{\;jr})\;$$

where *μ* is the total edge weight of the network, *A_ijl_* is the edge between nodes *i* and *j* at layer *l* of the multilayer network, and *V_ijl_* describes the corresponding element of a null model. The parameter *γ_l_* sets the structural resolution parameter of layer *l* (i.e. the weight of intralayer edges), the parameter *ω_jlr_* sets the temporal resolution parameter (i.e. the weight of interlayer edges, here *γ_l_* = 1, *ω_jlr_* = 0.4),^43^ and the parameter *g* describes the community assignments of two nodes across the time domain, involving node *i* in layer *l* and node *j* in layer *r*. *δ* is a Kronecker delta function, where *δ*(*g_il_*, *g_jr_*) = 1 if *il* = *jr* and 0 otherwise.

Although the current network should be considered orderly and have interlayer links between sequential layers for nodes at the same position, the generalized Louvain algorithm has a stochastic nature, sometimes causing the instability of community assignments.^44^ We performed 100 iterations for each subject and calculated the mean to ensure the stability of the results, similar to an implementation used in a previous study.^35^ In addition, to illustrate methodologically the reproducibility of the results, we used default parameters (*γ* = 1 and *ω* = 1) to verify the effect of variation of the structural and temporal resolution parameters on the results.

### Recruitment and integration

We calculated two dynamic indicators to quantify the dynamic interactions of inter- or intra-networks: recruitment and integration. The recruitment coefficient describes the average probability that node *i* is in the same community as other nodes from its own network and is defined as:

(2) $$R_{i}^{N}\;=\frac{1}{m_{N}}\sum_{\;j\in N}\;P_{ij}$$

where *m_N_* is the size of network *N*, calculated as the number of nodes in *N*, and *P_ij_* corresponds to the relative frequency at which nodes *i* and *j* were assigned to the same community across the time domain, where *P_ij_* = 1 if nodes *i* and *j* are always in the same community and 0 otherwise. Therefore, a node with high recruitment tends to be associated with nodes from its own network in the time domain.

The integration coefficient describes the average probability that node *i* is in the same community as nodes from other networks and is calculated using the following equation:

(3) $$I_{i}^{N}\;=\frac{1}{K-m_{N}}\sum_{\;j\notin N}\;P_{ij}$$

where *K* is the total number of nodes. A node with high integration tends to be associated with nodes from other networks in the time domain.

### Multisite effect correction

Site effects on the modular coefficient were removed using the ComBat function available in MATLAB (https://github.com/Jfortin1/ComBatHarmonization) to account for site, collection time and data acquisition parameter variability across each of the data collections in ABIDE I. This approach has been shown to effectively account for scanner-related variance in multisite resting-state fMRI datasets.^45^ During Combat, only the diagnosis was treated as a biological variable of interest, and a parametric prior method was used in the empirical Bayes procedure.

### Statistical analysis

We defined the mean recruitment and integration coefficients of ROIs within each network as the network-level parameters. The primary statistical processes were performed at the network level, and we compare 45 parameters (36 integration and 9 recruitment coefficients) between two groups. The significance was determined using independent sample *t*-tests with the false discovery rate (FDR) correction (*q* < 0.05). In addition, for the significant network integration and recruitment coefficients, we expected further to determine which ROI-level parameters contribute to these network variances. An independent sample *t*-tests (*P* < 0.001, uncorrected) was performed for ROI-level parameters to report these survival results.

### Data availability

The data stored at our lab-based network attachment system: http://QuickConnect.cn/others. ID: guests; PIN dong@123.COM.

## Results

### Recruitment and integration in functional networks

**Figure 2:**
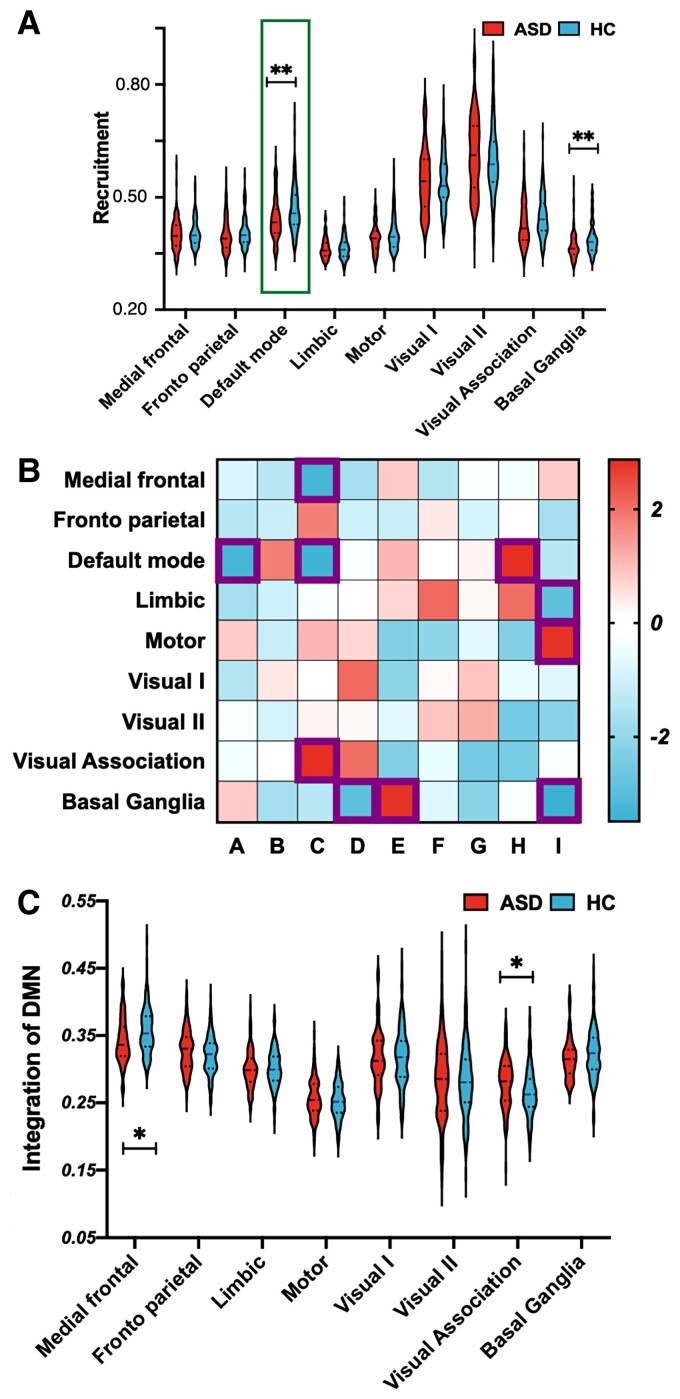
**Group differences in recruitment and integration coefficients in the DMN**. (**A**) Group difference (*t*-test) in the recruitment coefficient were observed in DNM (*t* = −3.3993, *P* = 0.0008) and BGN (*t* = −3.4847, *P* = 0.0006). (**B**) The integrations among networks. (**C**) When using the DMN as network of interest, we observed group differences (*t*-test) in DMN–MFG (*t* = −3.151, *P* = 0.0011) and DMN–visual association network (*t* = 2.8709, *P* = 0.0045) integration.

At recruitment, subjects with ASD showed a lower recruitment coefficient within the DMN than HCs (*t* = −3.3993, *q* = 0.0266, FDR correction, *P* = 0.0008, see Fig. 2A). The integration among networks is shown in Fig. 2B. When setting the DMN as the network of interest, we observed that subjects with ASD showed lower integration between the DMN and MFN (*t* = −3.3151, *q* = 0.0266, FDR correction, *P* = 0.0011) and between the DMN and visual association network (*t* = 2.8709, *q* = 0.0463, FDR correction, *P* = 0.0045, see Fig. 2C).

**Figure 3:**
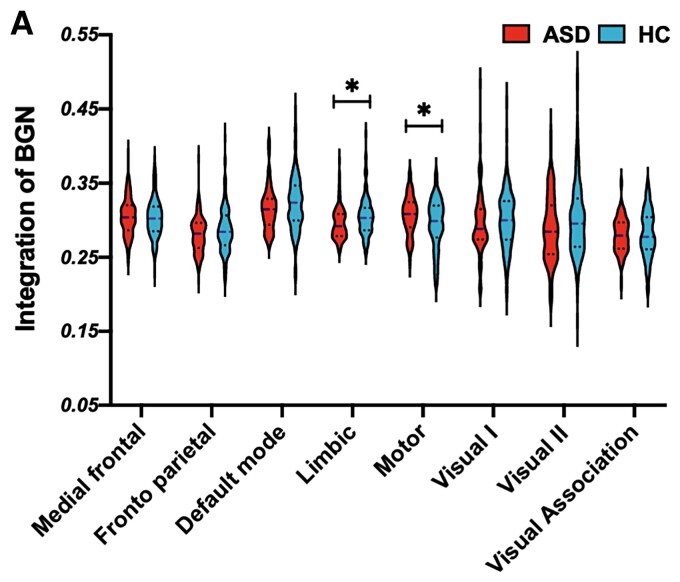
**Group differences in recruitment and integration coefficients in the BGN**. (**A**) When using the BGN as the network of interest, we observed group differences (*t*-test) in the BGN–limbic (*t* = −2.8996, *P* = 0.0041) and BGN–motor networks (*t* = −2.8053, *P* = 0.0055).

In addition to the DMN, a significant group difference was also observed in the BGN. At recruitment, subjects with ASD showed a lower recruitment coefficient within the BGN than HCs (*t* = −3.8467, *q* = 0.0266, FDR correction, *P* = 0.0006, see Fig. 2A). For the integration analysis, we set the BGN as the network of interest and observed that subjects with ASD showed lower integration between the BGN and limbic network (*t* = −2.8996, *q* = 0.0463, FDR correction, *P* = 0.0041) and between the BGN and motor network (*t* = −2.8053, *q* = 0.0463, FDR correction, *P* = 0.0055, see Fig. 3A). These findings were also well validated under default structural and temporal resolution parameters, supporting the reliability of the results (see Supplementary Table S1).

### Replication test with independent data

**Figure 4:**
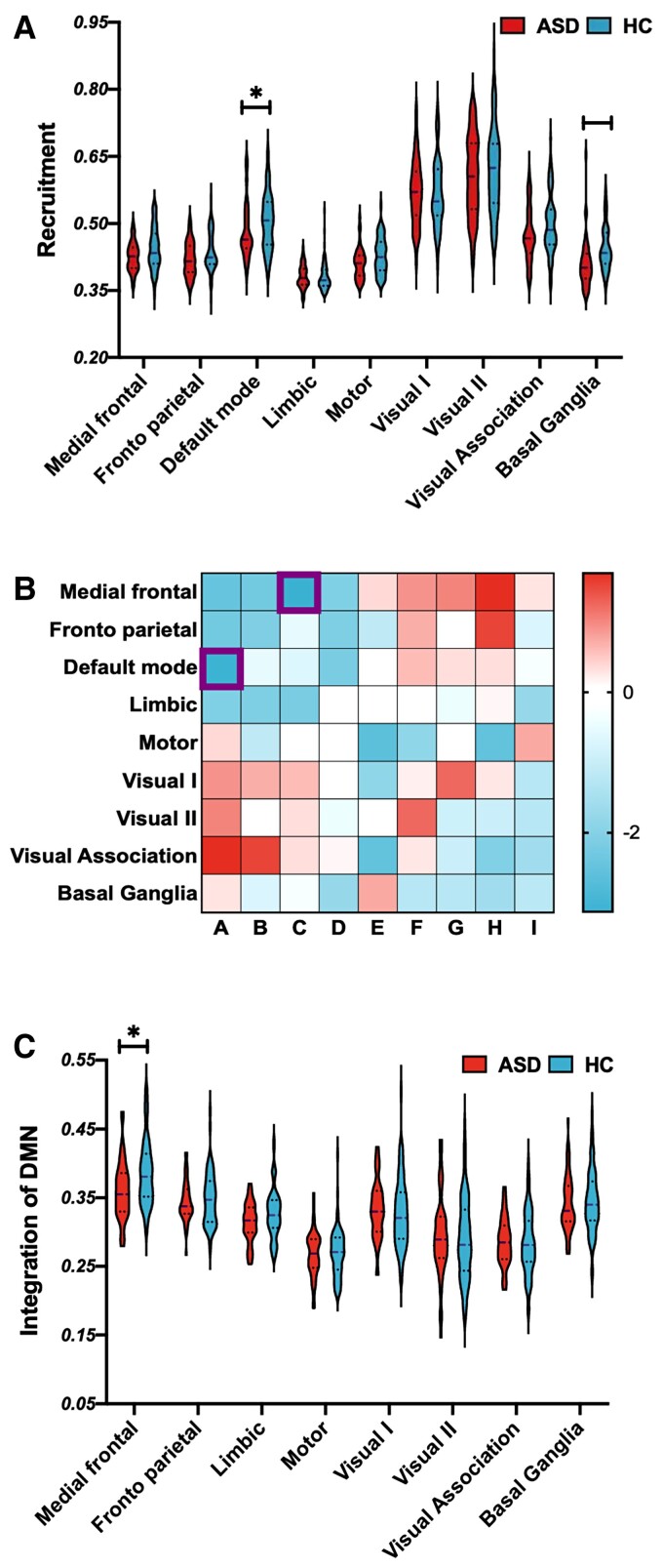
**Replication test with the ABIDE II database**. (**A**) Group difference (*t*-test) in the recruitment coefficient were observed in DMN (*t* = −3.51, *P* = 0.0006) but not in BGN (*t* = −1.21, *P* = 0.3224). (**B**) The integrations among networks. (**C**) When using the DMN as the network of interest, we observed group differences (*t*-test) in the DMN–MFG (*t* = −2.98, *P* = 0.0033) integration.

We tested the results with independent data (ABIDE II). All methods for preprocessing, network construction and statistical analyses were the same as the main process. At recruitment, subjects with ASD show a lower value in the DMN network than HCs (*t* = −3.1256, *P* = 0.0315) (Fig. 4A). Specifically, subjects with ASD showed lower DMN–MFN integration than HCs (*t* = −2.9538, *P* = 0.0374) (Fig. 4C).

However, in the BGN, group differences in recruitment were observed (*t* = −2.3516, *P* = 0.1024), but the differences did not reach statistical significance (Fig. 4A). Additionally, in terms of integration, no significant group difference was observed in the BGN (Fig. 4B). For the DMN, the different measures show very replicable results; however, for the BGN, the results were only observed in ABIDE I but not in ABIDE II (potential reasons are discussed in Discussion).

### Dynamic changes in functional features in different age groups

**Figure 5:**
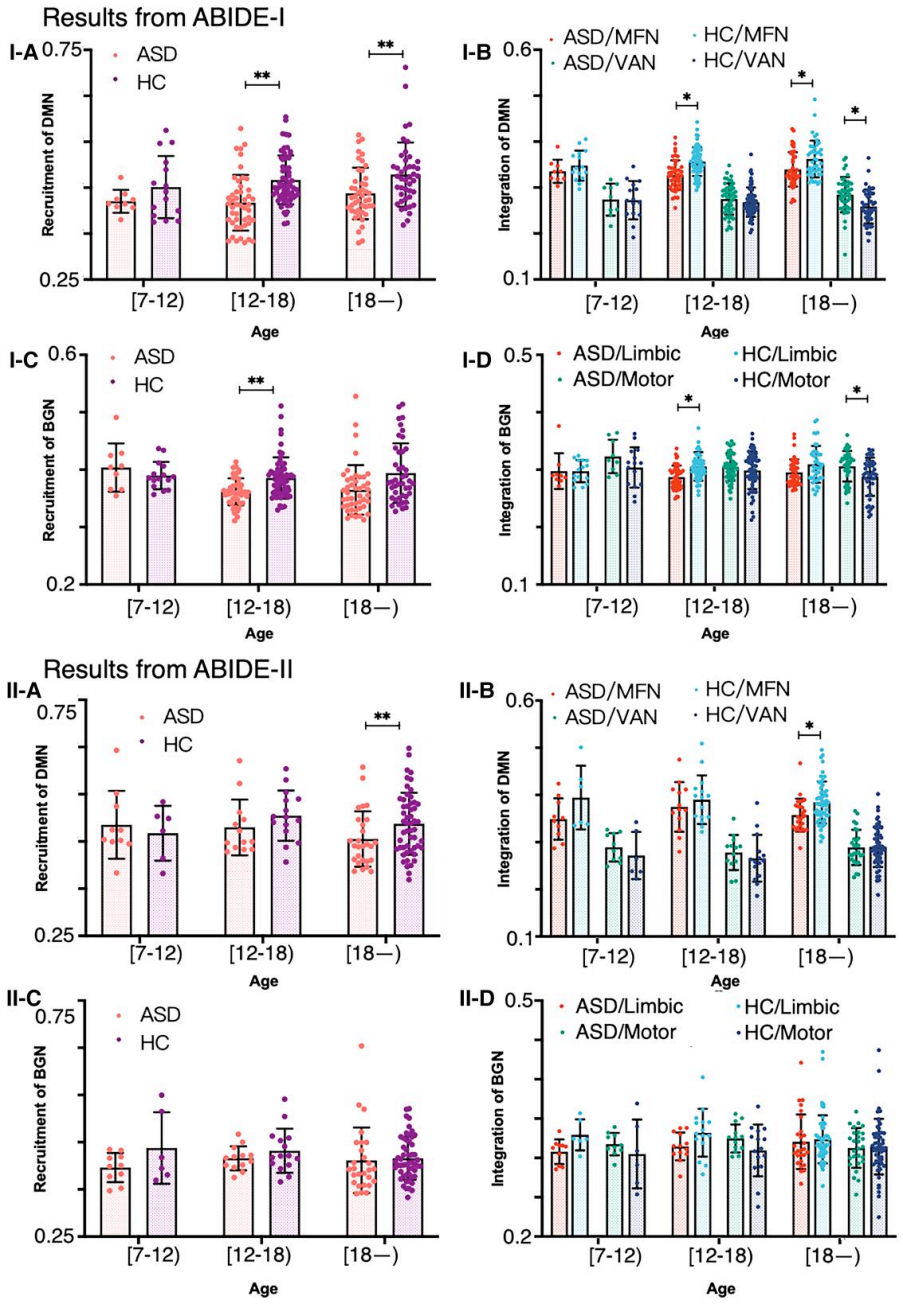
**Recruitment and integration features in different age groups and in different databases**. (**I-A**) Group difference in the recruitment coefficient for the DMN in 12–18 (*t* = −3.62, *P* = 0.0011) and 18–25 (*t* = −3.41, *P* = 0.0015) age groups. (**I-B**) The DMN was used as the network of interest to analyse the integration in different comparison groups. (**I-C**) Group difference in the recruitment coefficient in the BGN in 12–18 (*t* = −3.38, *P* = 0.0015) age groups. (**I-D**) The BGN was used as the network of interest to analyse the integration in different comparison groups. (**II-A–D**) The results that were tested in the ABIDE II dataset. All group differences were tested with *t*-test.

We divided subjects into three age groups: primary school age of 7–12 (not included) years, adolescents aged 12–18 (not included) years, and young adults aged 18–25 years. In the DMN, recruitment (ASD < HC) was observed in the 12–18- and 18–25-year-old age groups (Fig. 5I-A). A similar feature was also observed in ABIDE II, although the group difference did not reach statistical significance in the 12–18-year-old age group (Fig. 5I-A). In terms of integration in the DMN, subjects with ASD showed lower DMN–MFN integration in the 12–18-year-old and 18–25-year-old groups (Fig. 5I-B), and this feature was replicated in ABIDE II (Fig. 5I-B).

Subjects showed a significantly lower recruitment coefficient for the BGN in the 12–18-year-old age group and a trend in the adult group (although the results did not reach statistical significance) (Fig. 5I-C). However, these features were not observed in ABIDE II (Fig. 5I-C). Subjects with ASD showed a lower coefficient for integration in the BGN–limbic network in the 12–18-year-old age group and a higher integration coefficient in the BGN–motor network in the adult group (Fig. 5I-D). However, none of these recruitment and integration features was replicated in the ABIDE II database (Fig. 5I-D).

In order to examine whether age-effect regression results in spurious results towards age-dependent group differences. We re-analysed the results of age group and retained the age effects during the ComBat process. The detailed age-site distribution could be observed in Supplementary Fig. S2. Validation analysis demonstrated the robustness of the results, see Supplementary Fig. S3 for details.

## Discussion

Using dynamic network analyses, the current study provides a new perspective on the dynamic reconfiguration of the functional brain network in subjects with ASD. The current study found a disturbed community structure (recruitment and integration) in subjects with ASD, which might explain why subjects with ASD displayed the observed network features and cognitive functions.

### Subjects with ASD show altered recruitment and integration in the DMN compared with HCs

At recruitment, subjects with ASD showed a lower coefficient than HCs in the DMN. Recruitment refers to the probability that a brain region is in the same community as other nodes from its own network.^35^ In general, a lower recruitment coefficient indicates that the nodes within the network are less likely to be incorporated in the same community over time. The DMN is a large-scale network system in which regions interact instantaneously with sensory, motor and emotional systems to perform functions including autobiographical recall, imagining the future, making social and emotional judgments about oneself and others,^46^ making moral judgments and performing theory of mind tasks.^47^ It is closely related to self-related mental activity or generating mental simulations of the world^48^ and is of obvious relevance to psychiatric disorders, especially autism.^49,50^ DMN functional pathology is a factor contributing to the social cognitive impairment in individuals with ASD. Available evidence has revealed abnormalities in the DMN of people with ASD.^50^ Xie *et al*.^33^ reported that individuals with ASD showed a higher module switching in ASD individuals, involving the medial prefrontal cortex, posterior cingulate gyrus and angular gyrus, and inferred involvement of the DMN, which was consistent with our findings. The current results of poor DMN recruitment suggest that the functional DMN in subjects with ASD was decoupled from the dynamic process, suggesting an abnormal interrelationship between regions within the DMN.

In the integration analysis, when we set the DMN as the network of interest, subjects with ASD showed lower integration coefficients for the DMN–MFN and DMN–visual association network. Integration refers to the probability that a brain region is in the same community as nodes from other networks.^35^ A lower integration coefficient usually indicates that pairs of nodes (where one region of the pair is located in one system and the other region of the pair is located in the other system) are less frequently classified in the same module across layers.^51^ The MFN has been suggested to be responsible for executive control,^52,53^ and deactivation between the frontal cortex and DMN is observed in patients with different disorders. For example, in individuals with mania, studies observed reduced prefrontal activation and the failure of deactivation with the parietal cortex.^54^ Individuals with borderline personality disorder exhibit a failure of deactivation affecting the medial frontal cortex and the precuneus.^49^ In terms of poststroke depression symptoms, the DMN was functionally integrated with some core hubs, such as the dorsal prefrontal cortex.^55^ These features were also observed in subjects with ASD; for example, a study found a voxelwise correlation between posterior cingulate cortex and medial prefrontal cortex seeds and the whole brain in the ASD group.^56^ ‘Integration’ refers to the probability of intercommunication with regions from other subsystems,^17,18^ which usually suggests cognitive flexibility in cognitive tasks.^35^ Thus, the decreased measures of communication between the DMN and MFN provide insights into this association, suggesting that the DMN of participants with ASD exhibits impaired communication with executive control-related brain regions, which might hinder their control over DMN-related cognitive functions. This finding also provides potential insights into the network-based pathogenesis of ASD.

Taken together, we argue that these findings are complementary with previous studies, and the current results provide strong statistical inferences for network exploration and illustrate the unique inter-network interactions in ASD, involving the decreased decoupling with the DMN network and impaired flexibility among networks. These changes might hinder the brains of individuals with ASD from performing higher-level functions, integrating higher-level information, and differentiating and processing specific information.

### Subjects with ASD show lower recruitment and integration in the BGN than HCs

In addition to the DMN, subjects with ASD showed a lower BGN recruitment coefficient than HC subjects. As the core of the reward circuit, the BGN has been explored in many studies. Studies have proven that subjects with ASD show a reduced preference for social stimuli, which might be caused by the lower reward value of social stimuli in these individuals.^57^ The reward circuit evaluates, regulates and reinforces appetitive behaviours through dopaminergic signalling and is a core brain system for processing reward value.^58^ Multiple studies suggested a link between an aberrant mesolimbic reward pathway and dysfunction in reciprocal social interactions using preclinical animal models of autism^59,60^ and reported reduced nucleus accumbens engagement during processing social stimuli in both children and adults with ASD using fMRI.^61,62^ According to the meaning of the recruitment coefficient, the current results of poor BGN recruitment might suggest that the reward network was decoupled from the dynamic process in subjects with ASD, suggesting an abnormal interrelationship between regions within the BGN that might be related to their lower preference for social communications.

In the integration analysis, subjects with ASD showed a decreased BGN–limbic network integration coefficient. The limbic network is also part of the mesolimbic pathway and is dedicated to the integration of visceral and emotional states with cognition and behaviour and has been proven to be altered in subjects with ASD.^63^ A study showed that both structural and functional circuit aberrations in the mesolimbic reward pathway are related to parent-reported measures of social interaction impairments in affected children.^19^ The integration results further supported the hypothesis that deficits in the mesolimbic reward pathway contribute to impaired social skills in children with autism. In summary, the current results first confirmed these results for the reward circuit in individuals with ASD, further provided an explanation for the impaired reward pathways in subjects with ASD and provided fundamental insights into the neurobiological features underlying reduced social interest in humans.

### Replication test

We performed analyses using the ABIDE I database and tested the replicability using the ABIDE II database to ensure that the conclusions of the current study are scientifically rigorous.

For the DMN, the recruitment feature and integration were very similar between ABIDE I and ABIDE II. Even when we divided the subjects into subgroups based on age, we also observed great similarities between these two datasets. These results suggest that the DMN results are highly replicable in different datasets.

However, for the BGN, we only observed a group difference in recruitment and integration in ABIDE I, and the results were not replicated in ABIDE II. Further age group analyses provided an explanation for this issue. The main group difference in the BNG was observed in the 12–18-year-old age group; however, a limited number of subjects were included in the 12–18-year-old group in the ABIDE II dataset. Thus, although similar BGN features were observed in individuals with ASD in the ABIDE II dataset, the results did not reach statistical significance.

### The integration and recruitment features in different age groups

When the subjects were divided into different age groups, the results revealed a lower recruitment coefficient for the DMN in subjects with ASD than HC subjects in the 12–18-year-old group and 18–25-year-old group. Regarding integration, subjects with ASD showed lower DMN–MFN integration in the 12–18-year-old and 18–25-year-old age groups. Most of these results were replicated in the ABIDE II dataset. Subjects with ASD showed a lower recruitment coefficient for the BGN than HC subjects in the 12–18-year-old group; although a difference was observed in the 18–25-year-old, it did not reach statistical significance. In terms of integration, similar to the analysis of all subjects, subjects with ASD showed a lower BGN–limbic network integration coefficient than HCs in the 12–18-year-old group. However, all these results were not replicated in the ABIDE II dataset.

Previous studies have reported an age difference in individuals with ASD.^64–66^ For example, a study observed significant differences in inter-network connectivity in children and adolescents with ASD compared with adults.^67^ In terms of development, a nonlinear decreasing trend (increasing and then decreasing) has been reported; specifically, the modularity of ASD increased from children to adolescents and then decreases from adolescents to adults.^68^ The results obtained from different age groups confirmed the recruitment and integration results observed in all subjects, which suggests that the results might also be observed in individuals at different ages. One important exception is that group differences in integration and recruitment were not observed in the 7–12-year-old age group, regardless of the DMN or BGN. This discrepancy might be caused by the limited number of subjects in this group. Future studies should recruit more subjects from this age group to validate the results.

Taken together, these results from different age groups are consistent with the results obtained for all subjects and are consistent with the modularity of ASD increasing from children to adolescents and then decreasing from adolescents to adults.^68^ The current results provide an explanation for the neural features underlying the developmental features of ASD.

### Limitations

Several limitations of this study must be noted. First, the current study used resting-state data, which lacked correlations between the severity of ASD and network metrics, including recruitment or integration coefficients. Second, the numbers of subjects in different age groups, especially 7–12 years in ABIDE II, are limited, and future studies should include more subjects in this age group.

## Conclusions

First, subjects with ASD showed lower recruitment coefficients in the DMN and BGN than HCs, suggesting that their functions were decoupled from dynamic processes, making them less efficient at recruiting these networks to perform relevant cognitive functions. Second, subjects with ASD showed lower DMN–MFG integration than HCs, suggesting their impaired executive control ability. Third, subjects with ASD showed lower BGN–limbic integration, which might explain their lack of interest in social communications. All these results revealed the mechanism underlying the impaired executive functions and social skills of subjects with ASD. These findings have important therapeutic implications for developing effective intervention strategies for subjects with ASD.

## Supplementary Material

fcac177_Supplementary_Data

## Abbreviations

ABIDE =: Autism Brain Imaging Data Exchange
ASD =: Autism spectrum disorder
BGN =: basal ganglia network
DMN =: default mode network
FDR =: false discovery rate
FPN =: frontoparietal network
HCs =: healthy controls
MFN =: medial frontal network
ROI =: regions of interest
