# Supplementary material for: Disrupted dynamic network reconfiguration of the brain functional networks of individuals with autism spectrum disorder
Source: Brain Commun. 2022 Aug 1;4(4):fcac177. doi: 10.1093/braincomms/fcac177 (PMC9356733; doi:10.1093/braincomms/fcac177)
Supplement: fcac177_Supplementary_Data [file fcac177_supplementary_data.docx]

**Supplementary files for:**

**Disrupted dynamic network reconfiguration of the brain functional networks of individuals with autism spectrum disorder**

**The exclusion criteria in selecting subjects**

The exclusion criteria for subjects were as follows: 1) without functional or structural images; 2) without handedness information or with mixed handedness; 3) without full-scale intelligence quotient (FIQ) information or FIQ smaller than 70 (Floris et al., 2021); 4) eye status, time of repetition (TR), slice number, or data matrix size different from those of most subjects within a site; we used 180 time points for all Stanford subjects; 5) time points different from those of most subjects within a site (at the Stanford site, there were 20 subjects with 240 time points (50%), 17 subjects had 180 time points (42.5%), one subject had 181 time points, two subjects had 238 time points, and we truncated all the functional data to 180 time points.); 6) severe artefacts and signal losses in functional images (by visual inspection); 7) scan duration less than 100 time points (Van Dijk et al., 2010); 8) head motion exceeding 3 mm or 3 degrees; 9) bad spatial normalization (by visual inspection); 10) scan cover less than 91% of the whole brain; 11) spatial correlation < 0.6 (a threshold defined by mean - 2SD) between each participant’s regional homogeneity (ReHo) map and the group mean ReHo map (Yan et al., 2019); 12) some subjects were excluded to ensure group matching for age, FIQ, mean framewise displacement (mFD)(Jenkinson, Bannister, Brady, & Smith, 2002) (*p* > 0.05, Two-sample t-test) and sex and handedness (*p* > 0.05, Chi-square test) in each centre; 13) at each step, any sites (UCLA_2 after poor spatial normalization, Caltech after inadequate cover) with less than 20 individual datasets were excluded; 14) Subjects’ data with less than 200 time points; (15) No matched subjects at that age (including: ASD subjects without matched HC, and HC without matched ASD).

**
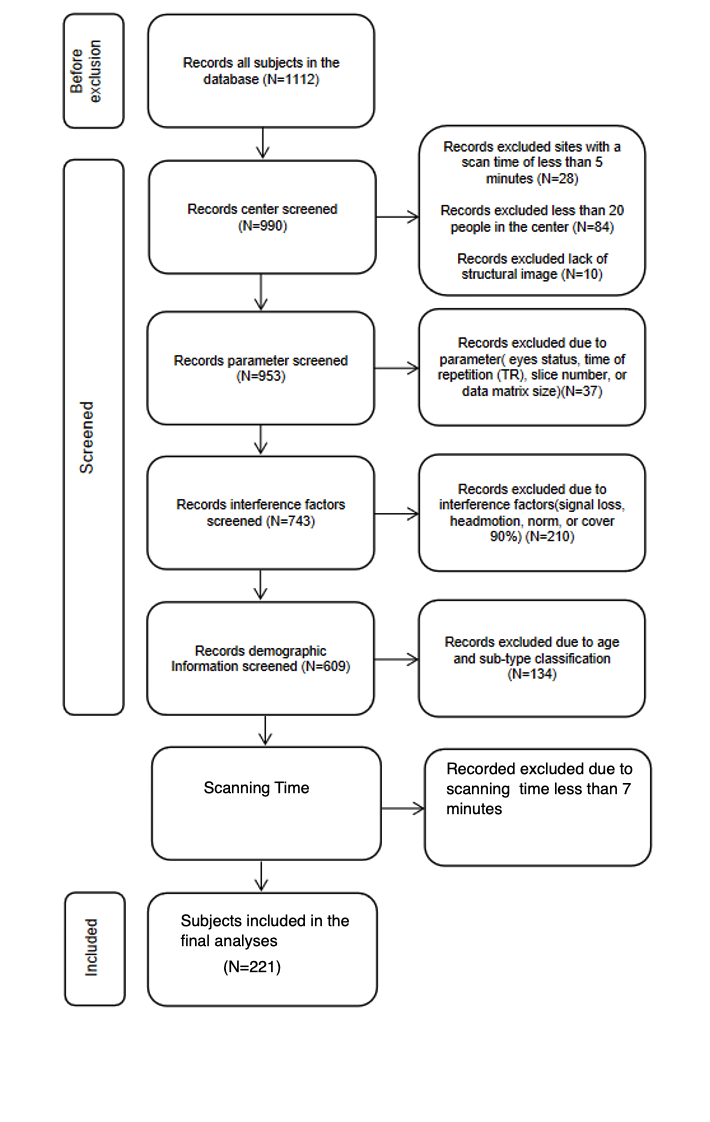
**

**Supplementary Fig. 1. Screening flowchart of the subject selection in current study**

The subplot shows that criteria for the screening of ABIDE-I and ABIDE-II database downloaded from the ABIDE webpage (Took ABIDE-I as an example).

Supplementary Table 1. Between-group differences using default parameters (γ =1 and ω=1)

|  | **ASD** | **HC** | ***t*** | ***FDR-q*** | ***p ^a^*** |
| --- | --- | --- | --- | --- | --- |
| ***Recruitment*** |  |  |  |  |  |
| DMN | *0.4477±0.0568* | *0.4752±0.0624* | *-3.3932* | *0.0315* | *0.0008* |
| BGN | *0.3761±0.0371* | *0.3946±0.0419* | *-3.4566* | *0.0315* | *0.0007* |
| ***Integration*** |  |  |  |  |  |
| DMN-MFN | *0.3473±0.0342* | *0.3635±0.0356* | *-3.4292* | *0.0315* | *0.0007* |
| BGN-Limbic | *0.3017±0.0222* | *0.3114±0.0274* | *-2.8458* | *0.0551* | *0.0049* |
| BGN-Motor | *0.3124±0.0262* | *0.3005±0.0343* | *2.8433* | *0.0551* | *0.0049* |

^a^ , uncorrected p value.

For the community parameter, we re-conducted the community detection using default parameter (γ=1 and ω=1). The results still support the previous findings: for the recruitment, subjects with ASD showed a lower recruitment coefficient within the DMN and the BGN than HCs (DMN: *t=-3.3932, q=0.0315*, FDR correction, *p=0.0008*; BGN: *t=-3.4566, q=0.0315*, FDR correction, *p=0.0007*). For the integration, subjects with ASD showed lower integration between the DMN and medial frontal network (*t=-3.4292, q=0.0315*, FDR correction, *p=0.0007*).

In addition, the parameters chosen in this study (the main manuscript) are γ=1 and ω=0.4, which has also been verified to be optimal by previous research.^20^ That research indicate that the results are robust to small variations in structural and temporal resolution parameter values, supporting the reliability of the findings.


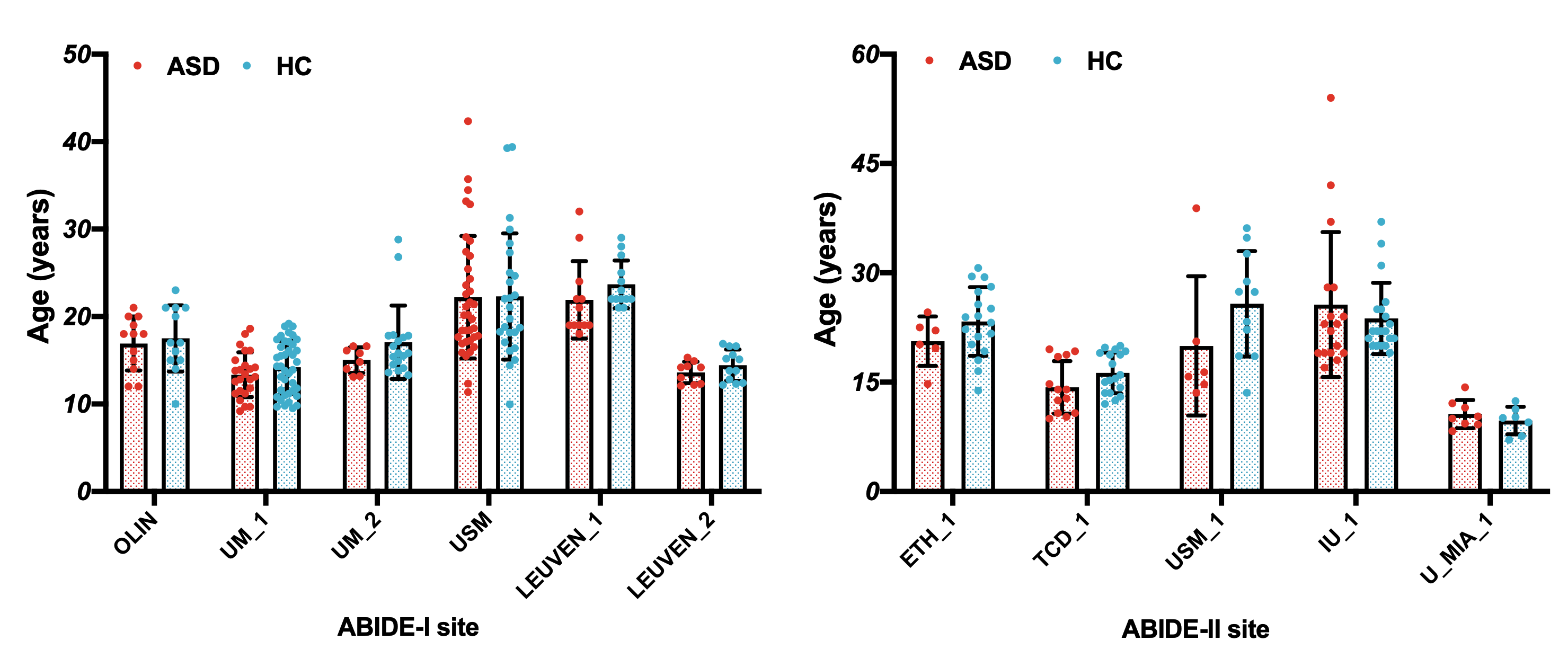


**Supplementary figure 2. The age-site distribution of participants in ABIDE-I and ABIDE-II**

Current selected participants from ABIDE I datasets involves 6 sites and ABIDE II datasets involves 5 sites. For each site, bright red circles denote ASD subjects and blue-green circles denote HC. OLIN, Olin, Institute of Living at Hartford Hospital; UM-1 and UM-2, University of Michigan: Sample 1and 2; USM, Utah School of Medicine; LEUVEN-1and LEUVEN-2, University of Leven: Sample 1and 2; ETH-1, ETH Zürich: Sample 1; TCD-1, Trinity Centre for Health Sciences: Sample 1; IU-1, Indiana University: Sample 1; U-MIA-1, University of Miami: Sample 1.

**
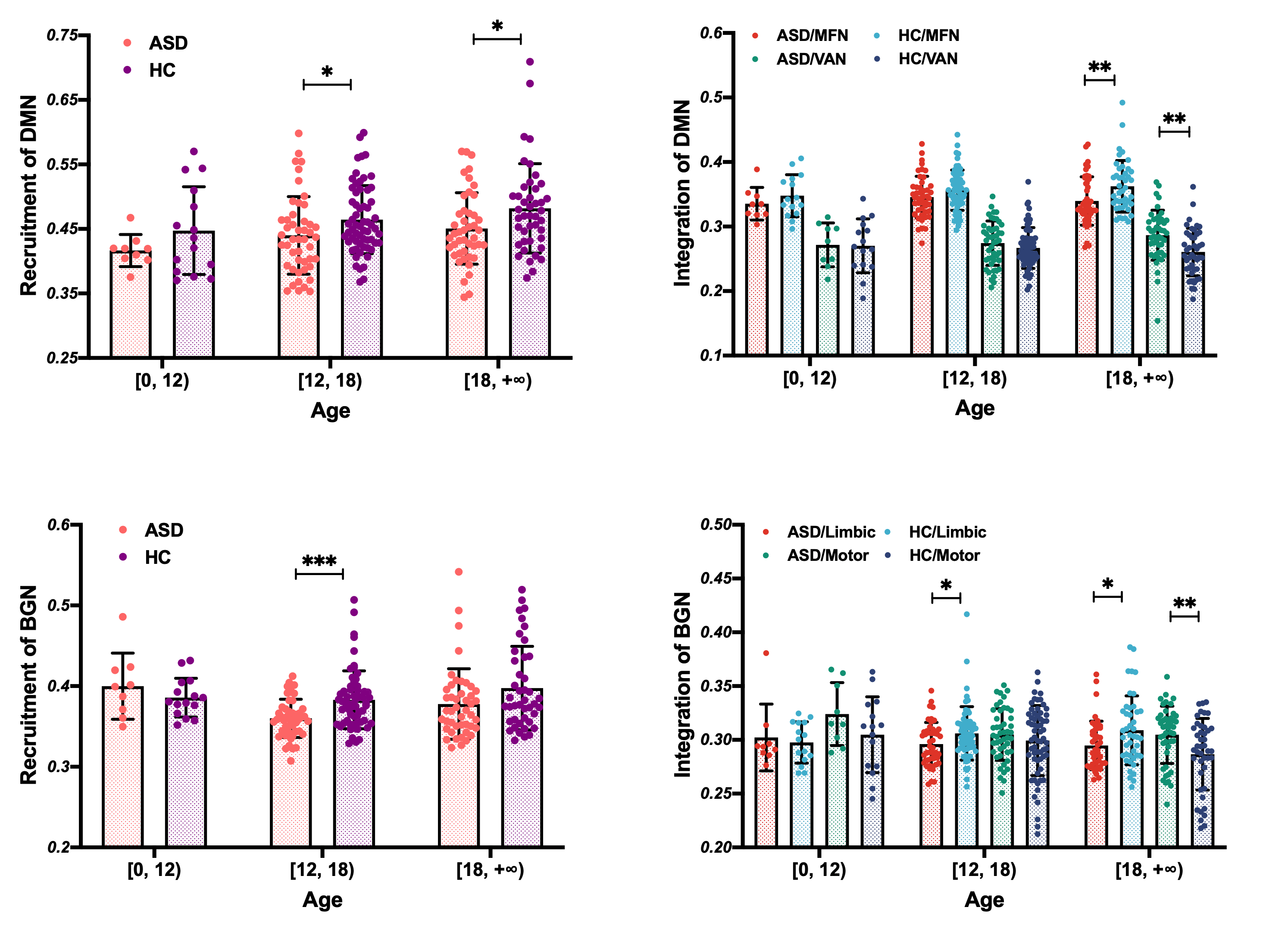
**

The effect of age was saved during ComBat process. Current between-group differences were similar to previous findings. *: <.05, **: <.01, ***:< .001.

Supplementary Figure 3. **Dynamic changes in functional features without age regression.**

In order to examine whether age-effect regression results in spurious results toward age-dependent group differences. We re-analyzed the results of age group and retained the age effects during the ComBat process. Validation analysis demonstrated the robustness of the results, see The above figures for details

Supplementary Table 2. The detailed information for subjects included in the current study

| Site | Muti-  ple  _site_  label | Sub  ID | Gr-  oup | DSM  -4 | AGE_  AT_  SCAN | SEX | Hand-  eness | FIQ | mFD | Time_  point | Medication  Name |
| --- | --- | --- | --- | --- | --- | --- | --- | --- | --- | --- | --- |
| OLIN | 1 | 50104 | 0 | 0 | 16 | 1 | 1 | 125 | 1.37E-01 | 200 |  |
| OLIN | 1 | 50105 | 0 | 0 | 17 | 1 | 1 | 100 | 7.14E-02 | 200 |  |
| OLIN | 1 | 50106 | 0 | 0 | 10 | 1 | 1 | 129 | 8.14E-02 | 200 |  |
| OLIN | 1 | 50107 | 0 | 0 | 21 | 1 | 1 | 132 | 6.84E-02 | 200 |  |
| OLIN | 1 | 50108 | 0 | 0 | 21 | 1 | 1 | 131 | 1.39E-01 | 200 |  |
| OLIN | 1 | 50111 | 0 | 0 | 14 | 1 | 1 | 118 | 2.09E-01 | 200 |  |
| OLIN | 1 | 50112 | 0 | 0 | 17 | 1 | 1 | 102 | 1.12E-01 | 200 |  |
| OLIN | 1 | 50113 | 0 | 0 | 15 | 0 | 1 | 100 | 1.39E-01 | 200 |  |
| OLIN | 1 | 50114 | 0 | 0 | 20 | 0 | 1 | 127 | 1.29E-01 | 200 |  |
| OLIN | 1 | 50115 | 0 | 0 | 23 | 1 | 2 | 135 | 1.40E-01 | 200 |  |
| OLIN | 1 | 50116 | 0 | 0 | 15 | 1 | 1 | 115 | 1.93E-01 | 200 |  |
| OLIN | 1 | 50117 | 0 | 0 | 21 | 1 | 2 | 135 | 1.15E-01 | 200 |  |
| OLIN | 1 | 50118 | 1 | 0 | 15 | 1 | 1 | 80 | 2.70E-01 | 200 | Aripiprazole; Venlafaxine |
| OLIN | 1 | 50119 | 1 | 0 | 19 | 0 | 1 | 132 | 8.14E-02 | 200 |  |
| OLIN | 1 | 50121 | 1 | 0 | 20 | 1 | 1 | 118 | 1.29E-01 | 200 |  |
| OLIN | 1 | 50122 | 1 | 0 | 12 | 1 | 2 | 112 | 6.82E-02 | 200 | Citalopram; Methylphenidate; Bupropion; Trazadone |
| OLIN | 1 | 50124 | 1 | 0 | 18 | 1 | 1 | 100 | 9.29E-02 | 200 |  |
| OLIN | 1 | 50127 | 1 | 0 | 14 | 0 | 1 | 115 | 1.16E-01 | 200 | Methylphenidate Extended Release; Methylphenidate |
| OLIN | 1 | 50128 | 1 | 0 | 16 | 1 | 1 | 114 | 8.74E-02 | 200 | Risperidone; Sertraline |
| OLIN | 1 | 50129 | 1 | 0 | 12 | 1 | 1 | 108 | 1.46E-01 | 200 | Oxcarbazepine; Sertraline; Methylphenidate Extended Release; Bupropion |
| OLIN | 1 | 50130 | 1 | 0 | 18 | 1 | 1 | 129 | 1.37E-01 | 200 | Methylphenidate; Methylphenidate Extended Release; Risperidone; Dextroamphetamine and Amphetamine |
| OLIN | 1 | 50131 | 1 | 0 | 21 | 1 | 2 | 71 | 1.22E-01 | 200 |  |
| OLIN | 1 | 50134 | 1 | 0 | 18 | 1 | 2 | 124 | 6.29E-02 | 200 |  |
| OLIN | 1 | 50137 | 1 | 0 | 20 | 1 | 1 | 120 | 2.07E-01 | 200 |  |
| UM_1 | 2 | 50272 | 1 | 1 | 14.2 | 1 | 1 | 98.5 | 8.78E-02 | 290 |  |
| UM_1 | 2 | 50273 | 1 | 1 | 16.8 | 1 | 1 | 112.5 | 4.64E-02 | 290 | Atomoxetine |
| UM_1 | 2 | 50284 | 1 | 2 | 11.2 | 0 | 1 | 111 | 1.33E-01 | 290 | Methylphenidate Extended Release |
| UM_1 | 2 | 50285 | 1 | 1 | 12.8 | 0 | 1 | 99.5 | 9.46E-02 | 290 |  |
| UM_1 | 2 | 50287 | 1 | 1 | 14.4 | 1 | 2 | 96.5 | 5.74E-02 | 290 | Risperidone; Peroxatine; Methylphenidate Extended Release |
| UM_1 | 2 | 50290 | 1 | 1 | 14 | 1 | 1 | 108.5 | 9.39E-02 | 290 |  |
| UM_1 | 2 | 50291 | 1 | 1 | 13.9 | 1 | 1 | 96 | 1.36E-01 | 290 |  |
| UM_1 | 2 | 50294 | 1 | 2 | 12.6 | 1 | 1 | 103 | 9.62E-02 | 290 |  |
| UM_1 | 2 | 50295 | 1 | 1 | 11.5 | 1 | 1 | 135 | 9.69E-02 | 290 |  |
| UM_1 | 2 | 50298 | 1 | 1 | 12.8 | 1 | 1 | 101 | 1.00E-01 | 290 | Fluoxetine |
| UM_1 | 2 | 50300 | 1 | 3 | 9.7 | 0 | 1 | 90 | 1.64E-01 | 290 |  |
| UM_1 | 2 | 50301 | 1 | 1 | 16.1 | 1 | 2 | 97 | 5.83E-02 | 290 | Fluoxetine; Atomoxetine |
| UM_1 | 2 | 50302 | 1 | -9999 | 10.4 | 0 | 1 | 97 | 1.66E-01 | 290 | Methylphenidate extended release |
| UM_1 | 2 | 50310 | 1 | 1 | 9.7 | 1 | 1 | 78.5 | 7.35E-02 | 290 |  |
| UM_1 | 2 | 50312 | 1 | 1 | 11.2 | 1 | 1 | 91 | 2.94E-01 | 290 | Atomoxetine |
| UM_1 | 2 | 50314 | 1 | 2 | 16.1 | 1 | 1 | 126 | 7.66E-02 | 290 |  |
| UM_1 | 2 | 50315 | 1 | 1 | 13.4 | 1 | 1 | 118.5 | 8.97E-02 | 290 |  |
| UM_1 | 2 | 50316 | 1 | 1 | 11.8 | 1 | 1 | 109 | 2.79E-01 | 290 |  |
| UM_1 | 2 | 50318 | 1 | 1 | 9.2 | 1 | 2 | 147.5 | 1.09E-01 | 290 |  |
| UM_1 | 2 | 50319 | 1 | 1 | 15 | 0 | 1 | 126 | 7.91E-02 | 290 |  |
| UM_1 | 2 | 50320 | 1 | 1 | 18.6 | 1 | 1 | 108 | 1.04E-01 | 290 | Paroxetine; Lisdexamfetamine |
| UM_1 | 2 | 50321 | 1 | 1 | 18 | 0 | 1 | 98.5 | 6.16E-02 | 290 | Aripiprazole; Synthroid |
| UM_1 | 2 | 50324 | 1 | 1 | 13.8 | 1 | 1 | 124.5 | 6.81E-02 | 290 |  |
| UM_1 | 2 | 50325 | 1 | 2 | 13.1 | 1 | 1 | 77 | 1.42E-01 | 290 | Methylphenidate extended release; Risperidone; Methylphenidate; Guanfacine |
| UM_1 | 2 | 50327 | 0 | 0 | 15.3 | 1 | 2 | 111 | 4.81E-02 | 290 |  |
| UM_1 | 2 | 50328 | 0 | 0 | 17.4 | 1 | 1 | 91.5 | 8.42E-02 | 290 |  |
| UM_1 | 2 | 50329 | 0 | 0 | 17.1 | 1 | 1 | 100 | 3.43E-02 | 290 |  |
| UM_1 | 2 | 50330 | 0 | 0 | 16.1 | 1 | 1 | 120 | 6.96E-02 | 290 |  |
| UM_1 | 2 | 50332 | 0 | 0 | 10.4 | 1 | 1 | 103.5 | 1.09E-01 | 290 |  |
| UM_1 | 2 | 50334 | 0 | 0 | 11 | 1 | 1 | 108 | 6.93E-02 | 290 |  |
| UM_1 | 2 | 50335 | 0 | 0 | 17.8 | 1 | 1 | 108 | 5.30E-02 | 290 |  |
| UM_1 | 2 | 50336 | 0 | 0 | 14.3 | 0 | 1 | 108 | 4.38E-02 | 290 |  |
| UM_1 | 2 | 50337 | 0 | 0 | 11.8 | 1 | 1 | 112 | 9.22E-02 | 290 |  |
| UM_1 | 2 | 50338 | 0 | 0 | 13.5 | 0 | 1 | 106.5 | 7.24E-02 | 290 |  |
| UM_1 | 2 | 50340 | 0 | 0 | 16.1 | 0 | 1 | 110 | 4.36E-02 | 290 |  |
| UM_1 | 2 | 50343 | 0 | 0 | 13.8 | 0 | 1 | 124.5 | 1.88E-01 | 290 |  |
| UM_1 | 2 | 50344 | 0 | 0 | 18.2 | 1 | 1 | 121.5 | 4.67E-02 | 290 |  |
| UM_1 | 2 | 50346 | 0 | 0 | 18.9 | 1 | 1 | 115.5 | 5.91E-02 | 290 |  |
| UM_1 | 2 | 50347 | 0 | 0 | 15.6 | 1 | 1 | 122 | 6.31E-02 | 290 | Levothyroxine |
| UM_1 | 2 | 50348 | 0 | 0 | 19.2 | 0 | 1 | 112.5 | 9.92E-02 | 290 |  |
| UM_1 | 2 | 50349 | 0 | 0 | 18.9 | 1 | 1 | 111 | 6.55E-02 | 290 |  |
| UM_1 | 2 | 50350 | 0 | 0 | 12.4 | 1 | 1 | 109.5 | 6.06E-02 | 290 |  |
| UM_1 | 2 | 50351 | 0 | 0 | 16.8 | 1 | 1 | 116 | 4.96E-02 | 290 |  |
| UM_1 | 2 | 50352 | 0 | 0 | 15.5 | 1 | 2 | 113.5 | 1.37E-01 | 290 |  |
| UM_1 | 2 | 50353 | 0 | 0 | 13.1 | 0 | 1 | 89 | 6.72E-02 | 290 |  |
| UM_1 | 2 | 50355 | 0 | 0 | 10.9 | 1 | 1 | 127.5 | 1.11E-01 | 290 |  |
| UM_1 | 2 | 50356 | 0 | 0 | 17.2 | 0 | 2 | 92.5 | 4.50E-02 | 290 |  |
| UM_1 | 2 | 50357 | 0 | 0 | 9.5 | 0 | 1 | 92.5 | 6.21E-02 | 290 |  |
| UM_1 | 2 | 50358 | 0 | 0 | 9.8 | 1 | 1 | 99.5 | 4.93E-02 | 290 |  |
| UM_1 | 2 | 50360 | 0 | 0 | 13.9 | 1 | 1 | 113.5 | 6.56E-02 | 290 |  |
| UM_1 | 2 | 50362 | 0 | 0 | 11.2 | 1 | 2 | 108 | 5.54E-02 | 290 |  |
| UM_1 | 2 | 50363 | 0 | 0 | 9.8 | 1 | 1 | 104.5 | 1.49E-01 | 290 |  |
| UM_1 | 2 | 50364 | 0 | 0 | 10.8 | 1 | 1 | 97 | 1.82E-01 | 290 |  |
| UM_1 | 2 | 50365 | 0 | 0 | 15.6 | 1 | 2 | 106.5 | 8.04E-02 | 290 |  |
| UM_1 | 2 | 50368 | 0 | 0 | 17.9 | 1 | 1 | 118.5 | 1.02E-01 | 290 |  |
| UM_1 | 2 | 50369 | 0 | 0 | 14.8 | 0 | 1 | 96.5 | 6.14E-02 | 290 |  |
| UM_1 | 2 | 50370 | 0 | 0 | 12.8 | 1 | 1 | 93.5 | 1.44E-01 | 290 |  |
| UM_1 | 2 | 50372 | 0 | 0 | 10.2 | 1 | 1 | 107.5 | 4.96E-02 | 290 |  |
| UM_1 | 2 | 50374 | 0 | 0 | 9.7 | 0 | 1 | 106 | 5.47E-02 | 290 |  |
| UM_1 | 2 | 50375 | 0 | 0 | 11.6 | 0 | 1 | 123.5 | 4.37E-02 | 290 |  |
| UM_1 | 2 | 50377 | 0 | 0 | 11.5 | 1 | 1 | 113.5 | 7.11E-02 | 290 |  |
| UM_1 | 2 | 50379 | 0 | 0 | 14.3 | 0 | 1 | 104 | 1.00E-01 | 290 |  |
| UM_1 | 2 | 50380 | 0 | 0 | 17.4 | 0 | 2 | 95.5 | 2.76E-01 | 290 |  |
| UM_1 | 2 | 50381 | 0 | 0 | 16.5 | 1 | 1 | 114.5 | 3.03E-01 | 290 |  |
| UM_2 | 3 | 50382 | 0 | 0 | 28.8 | 1 | 1 | 114 | 8.30E-02 | 290 |  |
| UM_2 | 3 | 50385 | 0 | 0 | 17.8 | 1 | 1 | 109 | 5.38E-01 | 290 |  |
| UM_2 | 3 | 50386 | 0 | 0 | 17.8 | 1 | 1 | 129 | 7.82E-02 | 290 |  |
| UM_2 | 3 | 50388 | 0 | 0 | 26.8 | 1 | 1 | 115 | 4.84E-02 | 290 |  |
| UM_2 | 3 | 50390 | 0 | 0 | 14.5 | 1 | 1 | 103.5 | 8.76E-02 | 290 |  |
| UM_2 | 3 | 50391 | 0 | 0 | 17.2 | 1 | 2 | 113.5 | 1.26E-01 | 290 |  |
| UM_2 | 3 | 50404 | 1 | 1 | 15.8 | 1 | 1 | 129.5 | 8.03E-02 | 290 | Methylphenidate extended release; Risperidone |
| UM_2 | 3 | 50405 | 1 | 1 | 16.6 | 1 | 1 | 94 | 1.15E-01 | 290 |  |
| UM_2 | 3 | 50407 | 1 | 1 | 16.6 | 0 | 1 | 105 | 4.52E-02 | 290 |  |
| UM_2 | 3 | 50408 | 1 | 1 | 14.8 | 1 | 1 | 118.5 | 9.39E-02 | 290 | Methylphenidate extended release |
| UM_2 | 3 | 50410 | 1 | 1 | 16.1 | 1 | 1 | 110.5 | 4.26E-02 | 290 | Atomoxetine |
| UM_2 | 3 | 50411 | 1 | 2 | 14 | 1 | 1 | 125.5 | 2.86E-01 | 290 |  |
| UM_2 | 3 | 50412 | 1 | 1 | 13.2 | 1 | 1 | 133.5 | 5.27E-02 | 290 |  |
| UM_2 | 3 | 50413 | 1 | 1 | 13.1 | 1 | 2 | 114.5 | 6.86E-02 | 290 |  |
| UM_2 | 3 | 50414 | 0 | 0 | 13.8 | 0 | 1 | 109 | 1.67E-01 | 290 |  |
| UM_2 | 3 | 50415 | 0 | 0 | 17.6 | 1 | 1 | 111.5 | 6.41E-02 | 290 |  |
| UM_2 | 3 | 50417 | 0 | 0 | 13.3 | 1 | 2 | 121.5 | 7.61E-02 | 290 |  |
| UM_2 | 3 | 50418 | 0 | 0 | 14.9 | 1 | 1 | 112 | 1.67E-01 | 290 |  |
| UM_2 | 3 | 50419 | 0 | 0 | 15.8 | 1 | 1 | 111 | 2.15E-01 | 290 |  |
| UM_2 | 3 | 50421 | 0 | 0 | 15.4 | 1 | 1 | 116 | 9.21E-02 | 290 |  |
| UM_2 | 3 | 50423 | 0 | 0 | 14.1 | 1 | 1 | 111.5 | 1.15E-01 | 290 |  |
| UM_2 | 3 | 50424 | 0 | 0 | 17.9 | 1 | 1 | 125.5 | 6.53E-02 | 290 |  |
| UM_2 | 3 | 50425 | 0 | 0 | 16.6 | 1 | 1 | 112 | 8.79E-02 | 290 |  |
| UM_2 | 3 | 50426 | 0 | 0 | 13.6 | 1 | 1 | 113 | 1.08E-01 | 290 |  |
| UM_2 | 3 | 50427 | 0 | 0 | 15.8 | 1 | 1 | 96 | 4.60E-02 | 290 |  |
| UM_2 | 3 | 50428 | 0 | 0 | 15.5 | 1 | 1 | 89.5 | 1.01E-01 | 290 |  |
| USM | 4 | 50433 | 0 | 0 | 18.7379 | 1 | 2 | 100 | 4.03E-02 | 230 |  |
| USM | 4 | 50434 | 0 | 0 | 18.2615 | 1 | 1 | 93 | 7.66E-02 | 230 |  |
| USM | 4 | 50438 | 0 | 0 | 15.0253 | 1 | 1 | 97 | 1.20E-01 | 230 |  |
| USM | 4 | 50440 | 0 | 0 | 23.948 | 1 | 1 | 98 | 8.22E-02 | 230 |  |
| USM | 4 | 50443 | 0 | 0 | 17.0705 | 1 | 1 | 120 | 9.24E-02 | 230 |  |
| USM | 4 | 50446 | 0 | 0 | 27.3238 | 1 | 1 | 116 | 9.29E-02 | 230 |  |
| USM | 4 | 50449 | 0 | 0 | 18.2177 | 1 | 1 | 108 | 6.84E-02 | 230 |  |
| USM | 4 | 50450 | 0 | 0 | 21.0979 | 1 | 1 | 95 | 1.77E-01 | 230 |  |
| USM | 4 | 50452 | 0 | 0 | 29.9685 | 1 | 1 | 112 | 4.55E-02 | 230 |  |
| USM | 4 | 50454 | 0 | 0 | 18.8008 | 1 | 1 | 111 | 5.23E-02 | 230 |  |
| USM | 4 | 50455 | 0 | 0 | 31.2827 | 1 | 1 | 112 | 1.46E-01 | 230 |  |
| USM | 4 | 50456 | 0 | 0 | 16.0986 | 1 | 2 | 109 | 1.68E-01 | 230 |  |
| USM | 4 | 50459 | 0 | 0 | 18.1547 | 1 | 1 | 111 | 4.94E-02 | 230 |  |
| USM | 4 | 50460 | 0 | 0 | 14.412 | 1 | 1 | 109 | 4.96E-02 | 230 |  |
| USM | 4 | 50461 | 0 | 0 | 16.3943 | 1 | 1 | 113 | 1.13E-01 | 230 |  |
| USM | 4 | 50462 | 0 | 0 | 24.6543 | 1 | 1 | 120 | 8.23E-02 | 230 |  |
| USM | 4 | 50463 | 0 | 0 | 28.3669 | 1 | 1 | 121 | 1.58E-01 | 230 |  |
| USM | 4 | 50464 | 0 | 0 | 9.9548 | 1 | 1 | 119 | 6.43E-02 | 230 |  |
| USM | 4 | 50466 | 0 | 0 | 39.2526 | 1 | 1 | 103 | 6.67E-02 | 230 |  |
| USM | 4 | 50467 | 0 | 0 | 19.7591 | 1 | 1 | 89 | 1.18E-01 | 230 |  |
| USM | 4 | 50468 | 0 | 0 | 39.3949 | 1 | 1 | 122 | 2.85E-02 | 230 |  |
| USM | 4 | 50471 | 0 | 0 | 25.0157 | 1 | 1 | 108 | 5.95E-02 | 230 |  |
| USM | 4 | 50472 | 0 | 0 | 22.1081 | 1 | 1 | 104 | 4.29E-02 | 230 |  |
| USM | 4 | 50473 | 0 | 0 | 22.4367 | 1 | 1 | 106 | 7.87E-02 | 230 |  |
| USM | 4 | 50474 | 0 | 0 | 22.0479 | 1 | 1 | 110 | 1.31E-01 | 230 |  |
| USM | 4 | 50475 | 1 | 1 | 21.6482 | 1 | 1 | 117 | 8.67E-02 | 230 |  |
| USM | 4 | 50476 | 1 | 1 | 20.1697 | 1 | 1 | 108 | 7.96E-02 | 230 |  |
| USM | 4 | 50477 | 1 | 1 | 20.1807 | 1 | 1 | 92 | 9.71E-02 | 230 |  |
| USM | 4 | 50479 | 1 | 1 | 22.8802 | 1 | 1 | 113 | 2.61E-01 | 230 |  |
| USM | 4 | 50480 | 1 | 1 | 29.0897 | 1 | 1 | 127 | 1.14E-01 | 230 |  |
| USM | 4 | 50482 | 1 | 1 | 27.4114 | 1 | 1 | 94 | 8.42E-02 | 230 |  |
| USM | 4 | 50484 | 1 | 1 | 19.7043 | 1 | 1 | 102 | 3.68E-02 | 230 |  |
| USM | 4 | 50485 | 1 | 1 | 23.5948 | 1 | 1 | 132 | 6.79E-02 | 230 |  |
| USM | 4 | 50486 | 1 | 1 | 17.7878 | 1 | 1 | 97 | 1.38E-01 | 230 |  |
| USM | 4 | 50491 | 1 | 1 | 26.9487 | 1 | 1 | 125 | 4.41E-02 | 230 |  |
| USM | 4 | 50493 | 1 | 1 | 35.7098 | 1 | 2 | 102 | 8.61E-02 | 230 |  |
| USM | 4 | 50495 | 1 | 1 | 22.5599 | 1 | 2 | 88 | 6.54E-02 | 230 |  |
| USM | 4 | 50496 | 1 | 1 | 24.3231 | 1 | 1 | 129 | 1.71E-01 | 230 |  |
| USM | 4 | 50497 | 1 | 1 | 34.4613 | 1 | 1 | 80 | 9.17E-02 | 230 |  |
| USM | 4 | 50498 | 1 | 1 | 21.1307 | 1 | 1 | 99 | 7.49E-02 | 230 |  |
| USM | 4 | 50499 | 1 | 1 | 25.4346 | 1 | 1 | 113 | 6.35E-02 | 230 |  |
| USM | 4 | 50500 | 1 | 1 | 17.2375 | 1 | 1 | 118 | 3.64E-02 | 230 |  |
| USM | 4 | 50503 | 1 | 1 | 28.6708 | 1 | 1 | 80 | 8.30E-02 | 230 |  |
| USM | 4 | 50504 | 1 | 1 | 17.6454 | 1 | 1 | 87 | 1.74E-01 | 230 |  |
| USM | 4 | 50505 | 1 | 1 | 33.1828 | 1 | 1 | 95 | 2.68E-02 | 230 |  |
| USM | 4 | 50506 | 1 | 1 | 15.9343 | 1 | 1 | 73 | 1.03E-01 | 230 |  |
| USM | 4 | 50512 | 1 | 1 | 15.4908 | 1 | 1 | 121 | 1.19E-01 | 230 |  |
| USM | 4 | 50513 | 1 | 1 | 18.4668 | 1 | 2 | 102 | 1.01E-01 | 230 |  |
| USM | 4 | 50514 | 1 | 1 | 21.4018 | 1 | 1 | 109 | 6.53E-02 | 230 |  |
| USM | 4 | 50515 | 1 | 1 | 15.8522 | 1 | 1 | 94 | 9.50E-02 | 230 |  |
| USM | 4 | 50516 | 1 | 1 | 17.1143 | 1 | 1 | 107 | 2.81E-02 | 230 |  |
| USM | 4 | 50517 | 1 | 1 | 16.501 | 1 | 2 | 109 | 5.39E-02 | 230 |  |
| USM | 4 | 50518 | 1 | 1 | 18.6585 | 1 | 2 | 77 | 4.94E-02 | 230 |  |
| USM | 4 | 50520 | 1 | 1 | 17.6728 | 1 | 1 | 76 | 1.63E-01 | 230 |  |
| USM | 4 | 50521 | 1 | 1 | 18.4175 | 1 | 1 | 80 | 1.70E-01 | 230 |  |
| USM | 4 | 50524 | 1 | 1 | 12.334 | 1 | 1 | 83 | 1.27E-01 | 230 |  |
| USM | 4 | 50525 | 1 | 1 | 32.8487 | 1 | 1 | 106 | 4.28E-02 | 230 |  |
| USM | 4 | 50527 | 1 | 3 | 18.4148 | 1 | 1 | 93 | 4.19E-02 | 230 |  |
| USM | 4 | 50528 | 1 | 1 | 11.3539 | 1 | 1 | 100 | 6.38E-02 | 230 |  |
| USM | 4 | 50529 | 1 | 1 | 42.3354 | 1 | 1 | 128 | 1.41E-01 | 230 |  |
| USM | 4 | 50532 | 1 | 1 | 16.9199 | 1 | 1 | 84 | 1.30E-01 | 230 |  |
| LEUVEN_1 | 5 | 50683 | 0 | 0 | 24 | 1 | 1 | 106 | 8.41E-02 | 240 |  |
| LEUVEN_1 | 5 | 50685 | 0 | 0 | 23 | 1 | 1 | 112 | 1.37E-01 | 240 |  |
| LEUVEN_1 | 5 | 50686 | 1 | 1 | 19 | 1 | 1 | 92 | 7.27E-02 | 240 |  |
| LEUVEN_1 | 5 | 50687 | 0 | 0 | 22 | 1 | 1 | 124 | 7.56E-02 | 240 |  |
| LEUVEN_1 | 5 | 50688 | 0 | 0 | 21 | 1 | 1 | 98 | 6.76E-02 | 240 |  |
| LEUVEN_1 | 5 | 50689 | 1 | 1 | 21 | 1 | 1 | 106 | 4.42E-02 | 240 |  |
| LEUVEN_1 | 5 | 50690 | 1 | 1 | 22 | 1 | 1 | 101 | 9.83E-02 | 240 |  |
| LEUVEN_1 | 5 | 50691 | 0 | 0 | 22 | 1 | 1 | 146 | 7.51E-02 | 240 |  |
| LEUVEN_1 | 5 | 50692 | 0 | 0 | 22 | 1 | 1 | 109 | 4.42E-02 | 240 |  |
| LEUVEN_1 | 5 | 50693 | 1 | 1 | 22 | 1 | 1 | 128 | 5.11E-02 | 240 |  |
| LEUVEN_1 | 5 | 50694 | 1 | 1 | 19 | 1 | 1 | 109 | 8.43E-02 | 240 |  |
| LEUVEN_1 | 5 | 50695 | 1 | 1 | 19 | 1 | 1 | 101 | 1.05E-01 | 240 |  |
| LEUVEN_1 | 5 | 50696 | 1 | 1 | 20 | 1 | 1 | 121 | 5.96E-02 | 240 | Fluoxetine Hcl; Bupropion |
| LEUVEN_1 | 5 | 50698 | 0 | 0 | 28 | 1 | 1 | 109 | 8.61E-02 | 240 |  |
| LEUVEN_1 | 5 | 50699 | 0 | 0 | 21 | 1 | 1 | 104 | 5.82E-02 | 240 |  |
| LEUVEN_1 | 5 | 50702 | 1 | 1 | 18 | 1 | 1 | 100 | 7.22E-02 | 240 | Methylphenidate; Risperidone |
| LEUVEN_1 | 5 | 50703 | 0 | 0 | 29 | 1 | 1 | 108 | 1.10E-01 | 240 |  |
| LEUVEN_1 | 5 | 50704 | 1 | 1 | 29 | 1 | 1 | 119 | 4.12E-02 | 240 | Lamotrigine; Lithium Carbonate |
| LEUVEN_1 | 5 | 50705 | 1 | 1 | 24 | 1 | 1 | 111 | 9.79E-02 | 240 |  |
| LEUVEN_1 | 5 | 50706 | 0 | 0 | 22 | 1 | 2 | 134 | 6.83E-02 | 240 |  |
| LEUVEN_1 | 5 | 50707 | 0 | 0 | 22 | 1 | 1 | 113 | 8.32E-02 | 240 |  |
| LEUVEN_1 | 5 | 50708 | 1 | 1 | 32 | 1 | 1 | 89 | 5.39E-02 | 240 | Benperidol |
| LEUVEN_1 | 5 | 50709 | 0 | 0 | 25 | 1 | 1 | 116 | 9.45E-02 | 240 |  |
| LEUVEN_1 | 5 | 50710 | 0 | 0 | 27 | 1 | 1 | 106 | 6.36E-02 | 240 |  |
| LEUVEN_1 | 5 | 50711 | 1 | 1 | 19 | 1 | 1 | 126 | 8.89E-02 | 240 |  |
| LEUVEN_2 | 6 | 50722 | 0 | 0 | 13.8 | 0 | 2 | 87.5 | 6.64E-02 | 240 |  |
| LEUVEN_2 | 6 | 50723 | 0 | 0 | 13.8 | 0 | 2 | 98.5 | 9.50E-02 | 240 |  |
| LEUVEN_2 | 6 | 50724 | 0 | 0 | 15.1 | 1 | 1 | 104.5 | 7.61E-02 | 240 |  |
| LEUVEN_2 | 6 | 50726 | 0 | 0 | 16.6 | 1 | 1 | 118 | 1.04E-01 | 240 |  |
| LEUVEN_2 | 6 | 50728 | 0 | 0 | 15.6 | 1 | 1 | 112.5 | 7.79E-02 | 240 |  |
| LEUVEN_2 | 6 | 50730 | 0 | 0 | 12.3 | 0 | 1 | 110 | 1.12E-01 | 240 |  |
| LEUVEN_2 | 6 | 50734 | 0 | 0 | 16.6 | 1 | 1 | 97 | 7.80E-02 | 240 |  |
| LEUVEN_2 | 6 | 50737 | 0 | 0 | 12.2 | 1 | 1 | 111 | 1.79E-01 | 240 |  |
| LEUVEN_2 | 6 | 50738 | 0 | 0 | 12.8 | 1 | 1 | 113.5 | 8.37E-02 | 240 |  |
| LEUVEN_2 | 6 | 50739 | 0 | 0 | 15.2 | 1 | 1 | 111 | 6.90E-02 | 240 |  |
| LEUVEN_2 | 6 | 50740 | 0 | 0 | 12.4 | 1 | 1 | 112 | 1.18E-01 | 240 |  |
| LEUVEN_2 | 6 | 50742 | 0 | 0 | 16.9 | 1 | 1 | 112.5 | 1.44E-01 | 240 |  |
| LEUVEN_2 | 6 | 50743 | 1 | 1 | 14.2 | 0 | 1 | 82.5 | 8.14E-02 | 240 |  |
| LEUVEN_2 | 6 | 50745 | 1 | 1 | 13 | 1 | 1 | 94.5 | 5.64E-02 | 240 |  |
| LEUVEN_2 | 6 | 50748 | 1 | 1 | 14.2 | 1 | 1 | 105.5 | 8.79E-02 | 240 |  |
| LEUVEN_2 | 6 | 50749 | 1 | 1 | 15.3 | 0 | 2 | 86 | 1.02E-01 | 240 |  |
| LEUVEN_2 | 6 | 50751 | 1 | 1 | 12.1 | 1 | 1 | 128 | 1.86E-01 | 240 |  |
| LEUVEN_2 | 6 | 50754 | 1 | 1 | 12.3 | 1 | 1 | 97 | 5.11E-02 | 240 |  |
| LEUVEN_2 | 6 | 50755 | 1 | 1 | 12.2 | 1 | 1 | 99.5 | 6.51E-02 | 240 |  |
| LEUVEN_2 | 6 | 50756 | 1 | 1 | 14.9 | 1 | 1 | 103 | 1.12E-01 | 240 |  |
| LEUVEN_2 | 6 | 50757 | 1 | 1 | 14.3 | 1 | 1 | 76.5 | 1.38E-01 | 240 |  |
